# The Contribution of Carotenoids, Phenolic Compounds, and Flavonoids to the Antioxidative Properties of Marine Microalgae Isolated from Mediterranean Morocco

**DOI:** 10.3390/molecules24224037

**Published:** 2019-11-07

**Authors:** Imane Haoujar, Francesco Cacciola, Jamal Abrini, Domenica Mangraviti, Daniele Giuffrida, Yassine Oulad El Majdoub, Ayoub Kounnoun, Natalizia Miceli, Maria Fernanda Taviano, Luigi Mondello, Francesca Rigano, Nadia Skali Senhaji

**Affiliations:** 1 Laboratory of Microbiology and Applied Biotechnology, Department of Biology, Faculty of Sciences of Tetouan, Abd Al-Malek Essaadi University, Tetouan 93000, Morocco; abrinij@hotmail.com (J.A.); a.kounnoun@gmail.com (A.K.); senhajin@hotmail.com (N.S.S.); 2 Department of Biomedical, Dental, Morphological and Functional Imaging Sciences, University of Messina, 98166 Messina, Italy; dgiuffrida@unime.it; 3 Department of Chemical, Biological, Pharmaceutical and Environmental Sciences, University of Messina, 98166 Messina, Italy; dmangraviti@unime.it (D.M.); yassine.ouladelmajdoub@edu.umi.ac.ma (Y.O.E.M.); mtaviano@unime.it (M.F.T.); lmondello@unime.it (L.M.); 4 Chromaleont s.r.l., c/o Department of Chemical, Biological, Pharmaceutical and Environmental Sciences, University of Messina, 98168 Messina, Italy; francesca.rigano@chromaleont.it; 5 Laboratory of Plant Biotechnologies and Molecular Biology, Department of Biology, Faculty of Sciences of Meknes, Moulay Ismail University, Meknes 50000, Morocco; 6 Unit of Food Science and Nutrition, Department of Medicine, University Campus Bio-Medico of Rome, 00128 Rome, Italy; 7 BeSeps.r.l., c/o Department of Chemical, Biological, Pharmaceutical and Environmental Sciences, University of Messina, 98168 Messina, Italy

**Keywords:** microalgae, antioxidants, phenolic, carotenoids, HPLC–PDA–MS

## Abstract

This study aimed to investigate the potential of four sea water microalgae, isolated and cultivated at M′diq Bay in Morocco, as a new source of natural antioxidants. These microalgae belong to different classes, including *Phaedactylium tricornitum* (Bacillariophyceae), *Nannochloropsis gaditana* (Eustigmatophyceae), *Nannochloris* sp (Trebouxiophyceae), and *Tetraselmis suecica* (Chlorodendrophycea). The antioxidant properties were screened by the use of in vitro assays, namely 2,2-difenil-1-picrylhydrazyl, Ferric reducing antioxidant power, and Ferrous ions chelating activity, and compoundidentification was carried out in methanol and acetone extracts of both dried and fresh microalgae biomass by HPLC–PDA–MS analysis. Among the investigated microalgae, *Phaedactylium tricornutum* was the richest one regarding its carotenoid (especially all-E-fucoxanthin) and phenolic (especially protocatechuic acid) contents, as well as antioxidant activity (65.5%), followed by *Nannochloris* sp, *Tetraselmis suicica*, and *Nannochloropsis gaditana,* with antioxidant activity of 56.8%, 54.9%, and 51.1%, respectively.

## 1. Introduction

Replacing antioxidant compounds from an artificial source with other natural sources has been the general trend in recent decades. Antioxidant compounds are used for many purposes, such as bioactive compounds in functional foods, or to increase food shelf life and prevent unwanted lipid oxidation. The majority of commercially available natural antioxidants are isolated from terrestrial plants [1]. Microalgae are considered one of the oldest living organisms on planet Earth, and they have the ability to develop in different environments, like the sea and desert [2]. In recent years, the use of algae as an alternative source of bioactive compounds, such as polyphenols and carotenoids, might help to maintain the stability of land-based crop production [3]. As the global market of food/nutraceutical supplements based on microalgae extracts is well developing and has significant growth potential, the exploration of natural antioxidant composition and antioxidant properties of novel microalgae biomass is gaining an ever increasing importance. To this regard, different studies on the evaluation of the antioxidant activity of specific species of microalgae such as *Phaeodactylum* species [4,5], *Nannochloropsis gaditana*, *Nannochloris* sp., and *Tetraselmis suecica,* have been reported [6].

Polyphenols are recognized as important natural antioxidants and include several thousand compounds with great diversity in structure, which can be divided into ten different main classes according to their basic chemical structure [7,8]. Polyphenols act as antioxidants by single electron transfer and by hydrogen atom transfer [9]. Some studies have shown that the content of phenolic substances in microalgae is less than or equal to the minimum amounts reported for terrestrial plants [10]. Few other studies relative to the characterization and identification of phenolic compounds in marine microalgae have been performed [9]. In a recent UPLC–MS/MS study, single phenolic compounds and hydroxycinnamic acids were determined in *Phaeodactylum tricornutum* and *Tetraselmis suecica* species [11]. In vivo and in vitro antioxidants tests concerning the screening of microalgae species have highlighted the potential of microalgae as a new source of safe antioxidants; a total of 32 samples of microalgae were screened to determine their antioxidant capacity using three different antioxidant assays: 1,1-diphenyl-2-picryl-hydrazil (DPPH) radical scavenging activity, Ferric reducing antioxidant power (FRAP), and ABTS radical scavenging capacity activity [9]. *Tetraselmis suecica* and *Phaeodactylum tricornutum* microalgae species are industrially-cultivated, exhibiting higher antioxidant activities, which could be evaluated as new potential sources of natural antioxidants. *Nannochloropsis gaditana* has has shown its antioxidant activity, indicating its potential use in nutritional and therapeutic applications [12].

Carotenoids are a family of yellow to orange–red terpenoidic pigments synthesized by photosynthetic organisms, as well as certain bacteria and fungi [13]. They areconsidered as antioxidants because of their deactivating and trapping free radicals capabilities, especially singlet oxygen quenching [9,14,15]. Carotenoids commonly include two classes, the first one is composed of hydrocarbon structures, generally named carotenes, and the second one is composed of structures containing oxygen atoms, called xanthophylls. Green microalgae, like plants, can synthesize xanthophylls, e.g., violaxanthin, antheraxanthin, zeaxanthin, neoxanthin, and lutein. However, many other additional xanthophylls, such as loroxanthin, astaxanthin, and canthaxanthin, are also describable. Brown algae or diatoms can produce diiatoxanthine, diadinoxanthin, and fucoxanthin [16]. Several studies have shown that carotenoids contribute significantly to the total antioxidant capacity of microalgae [9,10].

For a chemical characterization of locally isolated species of microalgae, the present study aimed to determine their polyphenolic and carotenoid content by HPLC–PDA–MS analysis and evaluate their antioxidant activity. Compound identification was carried out by using complementary data coming from PDA, MS, and literature data.

## 2. Results and Discussion

### 2.1. Extraction of Polyphenolic Compounds and Carotenoids

In order to decompose the cell walls with minimum risk of damage, an effective extraction method with a solvent capable of entering the cell and dissolving the targeted compounds must be followed [15,16].In the present study, carotenoids were evaluated in both the dried biomass and crude liquid samples during the exponential phase of the culture, and a method of extraction consisting of methanol and acetone mixture was employed.

### 2.2. Total Phenolics, Flavonoids, and Carotenoids and Antioxidant Activity

The results of the total phenolic content based on the Folin–Ciocalteu method ranged from 39.34 to 22.94 mg/g GAE, with statistically significant differences between the four species (Table 1); the highest and lowest concentrations were recorded in *P. tricornutum* and *N. gaditana*, respectively.

In order to get a broad overview of the antioxidant profile of the four algae samples investigated, three in vitro assays based on different mechanisms were conducted. The primary antioxidant properties were determined by the DPPH test, which is based on a combination of hydrogen atom transfer (HAT) and single electron transfer (SET) mechanisms, and the reducing power assay, recognized as SET-based method; the Fe^2+^ chelating activity assay was used to establish the secondary antioxidant ability [17,18,19]. The results of the tests are reported in Table 2.

Free radical reducing capacity assayed with 2,2-diphenyl-1-picrylhydrazyl test showed the presence of important antioxidant activity; 65.4, 56.8, 54.9, and 51.1% for *P. tricornutum, Nannochloris* sp, *T. suecica*, and *N. gaditana*, respectively. The highest radical scavenging activity was obtained of *Nannochloris* sp exhibiting IC_50_ value (356.00 µg/mL).

In the reducing power assay, the calculated ASE/mL values indicated that the activity decreased in the order BHT >*P. tricornutum* > *T. suecica* > *Nannochloris* sp > *N. gaditana.*

In the Fe^2+^ chelating activity assay, the activity decreased in the order EDTA > *T. suecica* > *N. gaditana* > *P. tricornutum* > *Nannochloris* sp.

The results of the antioxidant tests clearly indicate that all the extracts showed better secondary antioxidant properties than the primary ones, and that *T. suecica* exhibited the highest chelating efficacy.

In the present study, the highest phenolic content (39.39 mg/g GAE) and remarkable antioxidant activity (65.5%) were obtained in the microalgae *P. tricornutum* extract, compared with the other three microalgae extracts (Table 1). The highest phenolic content was found in *P. tricornutum* extract with 39.94 mg/g GAE, followed by *Nannochloris* sp and *T. suecica* with 33.23 and 28.03 mg/g GAE, respectively, while lowest phenolic content was found in *N. gaditana* extract with 22.94 mg/g GAE. Phenolic contents of the analyzed extracts were higher than those found in *P. tricornutum*(16.80 mg/g GAE) and *T. suecica* (25.5 mg/g GAE) isolated from Morocco [20]. On the other hand, these results are in agreement with the findings from Goiris et al. who reported the data on the polyphenolic content in industrially cultivated *P. tricornutum* and *T. suecica* species [9]. The polyphenolic contents in *N. gaditana* extract were lower than those found by Maadane et al. (2015) (32.0 mg/g GAE) and higher than those found by Goiris et al. (1.40 mg/g GAE) [9,20]. It is worth mentioning that the polyphenolic composition can substantially vary as a function of microalgae growth conditions (nutrients availability, temperature, stress application), and extracting solvents used for evaluation of the antioxidant activity [9,21]. Furthermore, in microalgae, the polyphenolic contents increase upon exposure to UV-light, suggesting that they indeed play a role in the antioxidative response to this type of stress [22,23].

For the other determination of total carotenoids content in the dried biomass of the investigated species, significant results were obtained with corresponding values of 5.63, 5.62, 5.14, and 3.34 mg/g determined in *Nannochloris* sp, *T. suecica*, *P. tricornutum*, and *N. gaditana*, respectively. In the crude liquid, a total carotenoid content was determined between 0.09 and 2.09 µg/mL in the four microalgae species. Our results are confirmed in other studies that showed a high accumulation of carotenoids can be achieved in different microalgae species [16,20,24,25,26].The results of Maadane et al. (2015) show a high accumulation of carotenoids in *Tetraselmis* sp.(4.6 mg/g), *Nannochloropsis gaditana* (3.0 mg/g), and *Phaeodactylum tricornitum* (6.3 mg/g) [20]. Goiris et al. 2012 also reported similar carotenoid contents in *Phaeodactylum tricornitum, Tetraselmis suecica,* and *Nannochloropsis* sp. with 6.14, 4.27, and 2.17 mg/g, respectively [9].

### 2.3. Determination of Phenolic Compounds in the Microalgae Species

#### 2.3.1. Identification of Phenolic Compounds

In the present study, the polyphenols in microalgae species were identified considering the available standard, retention time, UV-vis, and mass spectra (Table 3).

**Figure 1:**
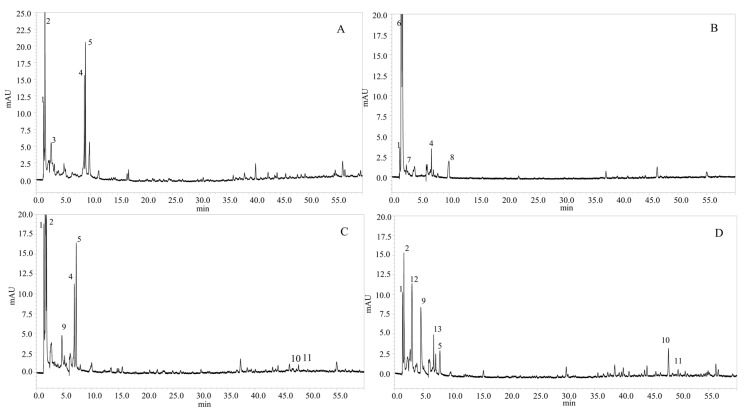
Chromatograms, obtained by HPLC–PDA–MS, of the polyphenolic compounds from four species of microalgae: (**A**) *Phaeodactylum tricornutum*, (**B**) *Nannochloris* sp, (**C**) *Tetraselmis suecica*, and (**D**) *Nannochloropsis gaditana*. Chromatographic conditions: See text. Peak identification is given in Table 3 (*λ* =280 nm).

Four classes of phenolic compounds were determined in the four species of microalgae (*P. tricornutum*, *T. suecica*, *Nannochloris* sp, and *N. gaditana*). Protocatechuic acid, which belongs to the hydroxybenzoic acid class, was determined at *m*/*z* 153 and UV-vis absorbance at *λ*_228–260_ nm, in all the microalgae samples (Figure 1); the same molecule was also reported to occur in *Jitai* plant [24]. Caffeic acid, which belongs to the class of hydroxycinnamic acid, was determined with a retention time of around 1.60 min in *P. tricornutum*, *T. suecica,* and *N. gaditana* species; this compound was determined also in the *Lamiaceae* spice plant [25]. In the same hydroxycinnamic acid class, caffeoyl glucoside was identified with *m*/*z* 341 in *T. suecica* and *N. gaditana* species, and p-coumaroyl tyrosine was identified in the negative mode at *m*/*z* 326 in *P. tricornutum*, *T. suecica*, and *N. gaditana* species with a retention time of 9.06 min, 7.27 min, and 7.18 min, respectively. Flavonoids represented the most abundant class with the highest number of polyphenolic compounds, such as dimethoxyflavone in *P. tricornutum*, *Nannochloris* sp, and *T. suecica*, rhamnosythexosyl-methyl-quercetin in *N. gaditana*, catechin at Rt 2.53 min with *m*/*z* 289 in *Nannochloris* sp, kaempferol at Rt 9.95 min with *m*/*z* 285 in *Nannochloris* sp, and quercetin at Rt 3.02 min with *m*/*z* 301 in *N. gaditana*.

**Figure 2:**
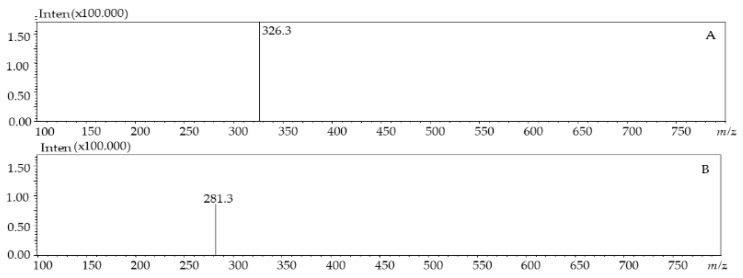
LC–MS (ESI^-^) profile ofp-coumaroyl tyrosine from *P. tricornutum* (**A**) and dimethoxyflavone from *T. suecica* species (**B**).

As an example, Figure 2 shows the MS spectra of p-coumaroyl tyrosine from *P. tricornutum* (A) and dimethoxyflavone from *T. suecica* (B) species.

#### 2.3.2. Quantification of Phenolic Compounds

Thirteenpolyphenols divided into four categories-hydroxycinnamic acid derivatives, hydroxybenzoic acid derivatives, hydroxycoumarin, and flavonoid—were quantified. Table 4 includes the molecular quantification in microgram/gram dry biomass of the four species (*P. tricornutum*, *Nannochloris* sp, *T. suecica*, and *N. gaditana*).

Protocatechuic acid, a subclass of hydroxybenzoic acid, was present in the four species of microalgae, with a maximum value of 40.55 μg/g dry biomass in *T. suecica* species. Caffeic acid, representing the hydroxycinnamic acid class, was present in three species (*P. tricornutum*, *T. suecica*, and *N. gaditana*) with the highest content of 59.53 μg/g of dry biomass determined in *T. suecica* species. In the same class, p-coumaroyl tyrosine was detected in three species, *T. suecica*, *P. tricornutum* and *N. gaditana*, with the highest value of 17.40 μg/g of dry biomass in *T. suecica* sample. For the flavonoids class, apigenin-O-rutinoside, catechin, and rhamnosylhexosyl-methyl-quercetin were quantified with contents of 35.75 μg/g in *T. suecica,* 33.47 μg/g of dry biomass in *Nannochloris* sp, and 6.89 μg/g in *N. gaditana*, respectively.

### 2.4. Determination of the Carotenoids in the Microalgae Species

#### 2.4.1. Identification of Carotenoids

**Figure 3:**
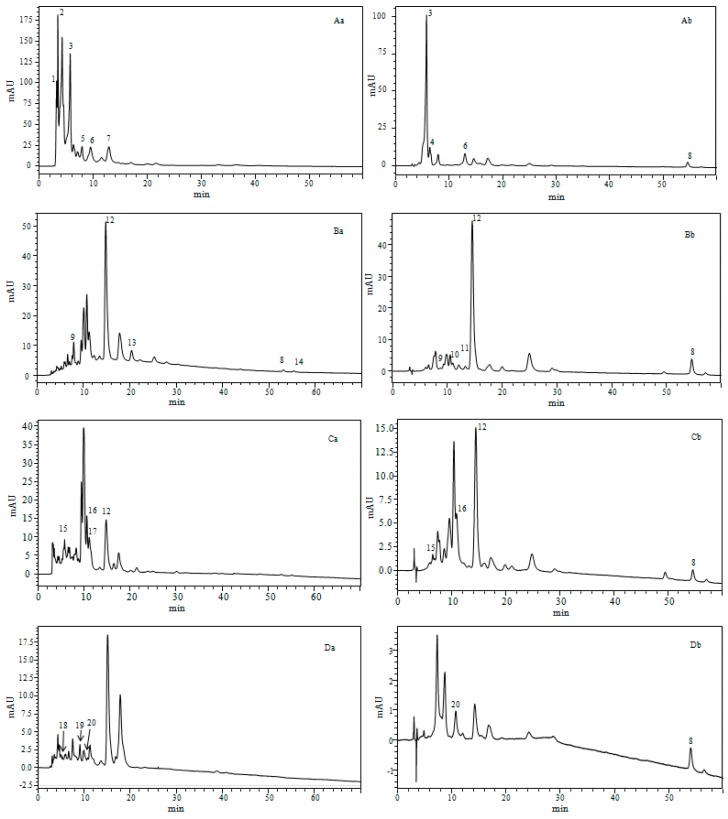
Chromatogram obtained by HPLC–PDA–MS (APCI) of the carotenoids for four species of microalgae: (**A**) *Phaeodactylum tricornutum*, (**B**) *Nannochloris* sp, (**C**) *Tetraselmis suecica*, and (**D**) *Nannochloropsis gaditana*. Chromatographic conditions: See text. With “a” for dry biomass and “b” crude liquid. Peak identification and characterization are given in Table 5 (*λ* = 450 nm).

The analysis of carotenoids from both the dried biomass and crude liquid of the four microalgae samples was carried out through a C30 column within 60 min; xanthophylls, chlorophylls, and hydrocarbon carotenoids were identified (Figure 3). LC–PDA–MS was employed for identification of carotenoids, listed in Table 5.

Based on the UV-vis and mass spectra interpretation, four different groups of carotenoids were determined: (i) Ketocarotenoids group represented by Echinone; (ii) hydroxy-carotenoids group expressed by vaucheriaxanthin, cis-15-lutein, cis-13-lutein, all-E-lutein, cis-prasinoxanthin, andzeaxanthin; (iii) carotenes represented by β-carotene and 9-cis-β-carotene; and (iv) epoxycarotenoids group characterized by six molecules—All-E-fucoxanthin, fucoxanthin isomers, diadinoxanthin, violaxanthin, neoxanthin, and antheraxanthin.

The qualitative and quantitative profiles were different among the investigated species, thus indicating that different metabolic pathways are prevailing in the different species; interestingly, this is the first work to report on the carotenoids characterization in crude liquid fractions (fresh microalgae), since only carotenoid content in dried biomasses were previously evaluated.

In our study, all-E-fucoxanthin (peak 3 Aa, 3 Ab) was the most abundant compound in *P. tricornutum*; a similar result was reported by Kim et al. in their study for the microalgae *Phaeodactylum tricornutum* [37]. Violaxanthin (peak 16 Ca, 16 Cb) was the abundant molecule in *T. suecica* species; a similar result was also reported in *Chlorella ellipsoidea* [38]. The most abundant carotenoid in *T. suecica* species was lutein. In *Nannochloris* sp Species, lutein (peak 12 Ba, 12 Bb) was the main carotenoid in both the dry biomass and crude liquid; lutein was also reported as the main carotenoid in *Phormidium autumnale* [39]. Zeaxanthin (peak 20 Da, 20 Db) was determined in *N. gaditana* species and xanthophyll was reported in *Phormidium autumnale* [39].

**Figure 4:**
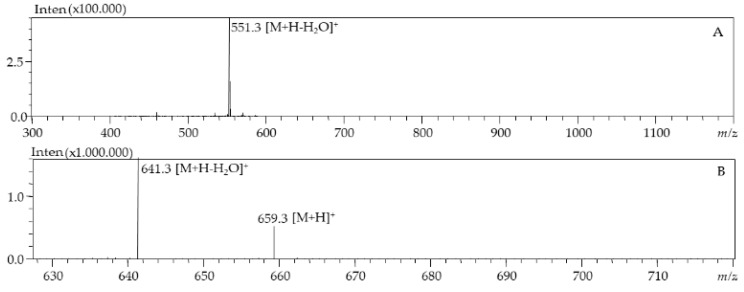
LC–MS (APCI^+^) profile of lutein from *Nannochloris* sp (**A**) and fucoxanthin from *P. tricornutum* species (**B**).

As an example, Figure 4 shows the MS spectra of lutein from *Nannochloris* sp and fucoxanthin from *P. tricornutum*, respectively.

#### 2.4.2. Quantification of Carotenoids in Four Marine Microalgae

The identified carotenoids in the microalgae samples were relatively quantified using the calibration curve of β-carotene. The highest total amount of carotenoids, quantified by HPLC–PDA, was obtained in *P. tricornutum* species with 60.90 µg/g and 1.73 µg/mL in dry biomass and crude liquid (fresh microalgae), respectively. In *Nannochloris* sp species, amounts of 12.50 µg/g and 0.94µg/mL were determined in dry biomass and crude liquid, respectively. In *T. suecica* dry biomass, an amount of 3.41 µg/g was evaluated, while for the crude liquid, the quantity was 0.23 µg/mL. In *N. gaditana*, the quantification values of dried biomass and crude liquid were lower than limit of quantification (LOQ). The spectrophotometric methods (Table 1) provided higher quantities of total carotenoids contents when compared to the HPLCUV-vis quantification values, probably because of the contributions from the unidentified carotenoids, and both carotenoid and chlorophyll degradation products [40,41]. Fucoxanthin was the most abundant carotenoid in *P. tricornutum* species (35.85 µg/g dry biomass), and it was reported to have an important role in photosynthesis [42]. Lutein was the next most abundant xanthophyll detected (12.50 µg/g dry biomass and 0.94 µg/mL crude liquid) in *Nannochloris* sp. Violaxanthin was determined in a concentration of 1.35 µg/g in the dry biomass in *T. suecica*; however, its concentration was lower when compared to *Chlorella* species [40], suggesting that *T. suecica* in the present study was not subjected to any external stress during growth phases. Zeaxanthin was detected, but at a concentration lower than the limit of quantification in *N. gaditana*; however, its low value indicates that the microalgae *N. gaditana* was not subjected to any stress, because zeaxanthin content in microalgae is regulated by light radiation [41].

Many studies have shown the role of carotenoids against several diseases, such as arteriosclerosis, cardiovascular diseases, cancer, and macular degeneration [43]. For example, lutein that was identified in *Nannochloris* sp and *T. suecica* species, and zeaxanthin in *N. gaditana*, play an essential role in the prevention of aging related to macular degeneration. The detected violaxanthin in *T. suecica* has shown a strong anti-proliferative activity capable of inhibiting the human mammary cancer cell line [43], and the detected fucoxanthin in *P. tricornutum* has been found to have several therapeutic activities, such as anticancer, antihypertensive, anti-inflammatory, and anti-obesity effects [44].

The four species of microalgae in this study showed the importance of possibly using them as new sources of bioactive ingredients in functional foods, pharmaceuticals, and nutraceuticals; therefore, the possible addition of these microalgae as food ingredients can offer health benefits to the consumer [45].

## 3. Materials and Methods

### 3.1. Chemicals and Reagents

Eight polyphenols standards (Gallic acid, Caffeic acid, Rutin, Catechin, Coumarin, Kaempferol, Apigenin, and Quercetin) and carotenoid standard(β-carotene) were obtained from Merck Life Science (Merck KGaA, Darmstadt, Germany). LC–MS grade methanol, acetonitrile, acetic acid, acetone, and water were purchased from Merck Life Science (Merck KGaA, Darmstadt, Germany).

### 3.2. Micro-Organism and Culture Conditions

**Figure 5:**
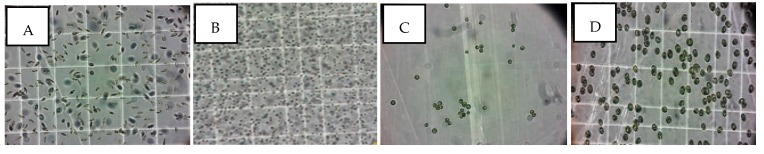
Light microscopic observation of isolated microalgae: (**A**) *Phaeodactylum tricornutum* (**B**) *Nannochloropsis gaditana*, (**C**) Nannochloris sp, and (**D**) *Tetraselmis suecica*.

Microalgae strains *Phaedactylum tricornitum* (P. *tri*), *Nannochloropsis gaditana* (N. *gad*), *Nannochloris* sp *KMMCC161* (N. *sp*), and *Tetraselmis suecica* (T. *sue*) were identified using morphological studies (Figure 5) and taxonomical approaches. The 18S rDNA, rbc 1, and rbc L primers’ molecular marker for each microalgae strain was amplified using 18SF, Euk516r, Tetra_rbcL_F, and Tetra_rbcL_R primers [46,47]. Sequencing of the amplified ribosomal genes was done by Pr. Manuel Manchado, Department Area de Cultivos Marinos y Recursos Pesqueros, Instituto de Investigación y Formación Agraria y Pesquera, Sevilla, Spain. An overview of the studied species, as well as their taxonomic classification, is given in Table 6.

*Phaeodactylum tricornutum* (MN625939), *Nannochloropsis gaditana* (MN625926), *Nannochloris* sp (MN625923), and *Tetraselmis suecica* (MN625941), which were deposited in the NCBI database under GenBank with mentioned accession numbers in parentheses, were stored at controlled conditions for preservation and future cultivation.

The culture conditions were as follows: An enriched air stream containing 5% of CO_2_ was passed through a water bottle from the bottom into the culture bottle. The culture medium (Table 7) was added in 1 L Erlenmeyer flasks, which were incubated in a controlled chamber, equipped with 16 fluorescent lamps (tubes). The temperature inside the culture chamber was controlled and was set at 21 °C. Stirring was insured by combining bubbling and magnetic stirring.

### 3.3. Sample Preparations

#### 3.3.1. Phenolic Compounds

First, 300 mg of each freeze-dried sample biomass was ground to a fine powder and added to 3 mL of pure methanol with vigorous agitation for 30 s. Then, the tubes were placed in a sonication bath (Elmasonic P 60 H, Konstanz, Germany) at room temperature for 45 min, and were centrifuged at 7500× *g* for 10 min. The extraction process was repeated three times in the same conditions. The collected supernatants were combined and stored at −20 °C. Finally, the methanolic extract solutions were evaporated and diluted with 1 mL of methanol and were filtered with a 0.22 µm PVDF syringe filter, the polyphenols and the flavonoids were analyzed by HPLC–PDA–(ESI)–MS.

#### 3.3.2. Carotenoids Contents

For the pigment analysis, dried biomass and crude liquid of microalgae were used. First, 200 mg of each lyophilized microalgae sample was extracted with 5 mL of acetone/methanol solvent mixture (7:3, *v*/*v*) and sonicated for 15 min. After evaporation at 35 °C, the solution was dissolved in methanol/methyl-tert-ether (MeOH/MTBE) (1:1, *v*/*v*), and was filtered with a 0.22 µm PTFE syringe filter. The same procedure was followed to evaluate the carotenoid contents in fresh microalgae samples by centrifuging 4 mLat 3000 rpm for 10 min. The filtrate was used immediately for carotenoid analysis by HPLC–PDA–(APCI)–MS [45].

### 3.4. Analytical Methods

#### 3.4.1. Antioxidant Properties

##### DPPH Radical Scavenging Activity Assay

DPPH radical scavenging activity assay was evaluated [48]. First, 1 mL of each microalgae extract was mixed with 1 mL of methanol, and then 2 mL of DPPH (0.004 g of DPPH in 100 mL of methanol) was added [49]. The mixtures were shaken vigorously and incubated at room temperature for 30 min in darkness. The absorption was determined spectrophotometrically at 517 nm. Ascorbic acid (Vit C) was used as standards. The IC_50_ (Inhibition Concentration at 50%) was used to compare the radical scavenging activity. The percentage of inhibition of DPPH (I%) was calculated using Equation (1):

I% = [(A simple blank−A extracted)/A blank] × 100(1)

A simple blank is the absorbance of extracts simple in methanol. A extracted is the absorbance of methanolic DPPH solution with the presence of all of the extract samples and standard. A blank is the absorbance of the methanolic DPPH solution.

##### Ferrous Ions Reduction Power (FRAP)

The reducing power of microalgae was evaluated by Fe^3+^–Fe^2+^ transformation method [19]. Different amounts of each extract in 1 mL methanol were mixed with 2.5 mL of phosphate buffer (0.2 M, pH 6.6) and 2.5 mL of 1% potassium ferrycyanide [K_3_Fe(CN)_6_], and incubated at 50 °C for 20 min. After being cooled down quickly, each sample was mixed with 2.5 mL of 10% trichloroacetic acid, and centrifuged at 3000 rpm for 10 min. A volume of 2.5 mL of the supernatant was mixed with 2.5 mL of distilled water and 0.5 mL of 0.1% fresh ferric chloride, and then the absorbance was measured at 700 nm after incubation in the dark at room temperature for 10 min. The increased absorbance of the reaction mixture indicates an increase in reducing power. Ascorbic acid and BHT were used as reference standards. The results were obtained from the average of three independent experiments, and are expressed as ascorbic acid equivalent (ASE/mL) ± SD.

##### Ferrous Ion-Chelating Ability

The Fe^2+^ chelating ability of microalgae was estimated by measuring the formation of the Fe^2+^–ferrozine complex [19]. Different concentrations of each extract in 1 mL methanol were mixed with 0.5 mL of methanol and 0.05 mL of 2 mM FeCl_2_; subsequently, 0.1 mL of 5 mM ferrozine was added to initiate the reaction. After shaking vigorously, the mixture was incubated in the dark at room temperature for 10 min; then, the absorbance of each sample solution was measured spectrophotometrically at 562 nm. The control contained FeCl_2_ and ferrozine, complex formation molecules. Ethylenediaminetetraacetic acid (EDTA) was used as reference standard. The results were obtained from the average of three independent experiments and are reported as IC_50_ ± SD.

#### 3.4.2. Phenolic Compounds Analysis

##### Total Phenolic Content

The total phenolic content of the four microalgae was determined by the Folin–Ciocalteu method [3]. Briefly, 200 μL of the diluted extract in ionized water (100 mg/mL) was mixed with 1 mL of Folin–Ciocalteu reagent in test tubes, and then 800 μL (75 g/L) of sodium carbonate was added. The samples were incubated in darkness for 30 min at room temperature, and then absorbance at 765 nm was measured by spectrophotometer (Rayleigh UV-Vis Spectrophotometer UV-1800). The total phenol content of the extracts is expressed in milligrams of Gallic acid equivalent (y = 0.011x − 0.002, R^2^ = 0.999).

##### Total Flavonoids

The total flavonoid content was evaluated by the colorimetric test [50]. First, 100 μg/mL of microalgae extract was added to 3 mL of methanol, and then 0.2 mL of potassium acetate 1 M, 0.2 mL of 10% aluminum chloride, and 5.6 mL of distilled water were added, respectively. The samples were incubated for 30 min in darkness at room temperature. The absorbance was measured at 415 nm using a UV spectrophotometer. The calibration curve was prepared by using quercetin standard in methanol, and the results are expressed with milligrams of quercetin equivalent per gram of sample (y = 0.0271x + 0.0129, R^2^ = 0.996).

##### Phenolic Compounds by HPL–-PDA–(ESI)–MS Analysis

The samples extracts were analyzed by a Nexera X2 liquid chromatography system (Shimadzu, Kyoto, Japan), consisting of a CBM-20A controller, two LC-30AD dual-plunger parallel flow pumps, and a DGU-20A5 degasser, and the analysis was performed on an Ascentis Express RP C18 column (2.7 µm, 150 mm, and 4.6 mm) (Merck Life Science, Merck KGaA, Darmstadt, Germany). The mobile phase consisted of water/acetic acid (99.85/0.15 *v*/*v*, solvent A) and acetonitrile (solvent B), with the following gradient elution: 0–5 min, 5% B, 5–15 min, 10% B, 15–30 min, 20% B, 30–60 min, 50% B, 60 min, 100% B. Photodiode array detector (PDA) was used online in the acquisition range of *λ*_200–400_ nm, where polyphenols were detected at *λ*_280_ nm and at *λ*_330_ nm (sampling frequency: 40 Hz, time constant: 0.08 s). The LC flow was 1 mL min^−1^ and mass spectra were obtained by an online Shimadzu mass spectrometer (LC-qMS-2020 Shimadzu), which was connected to an ESI source. MS analyses were performed in negative and positive modes, the scan range was set at *m*/*z* 100–800, and the scan speed was set at 2500 u sec^−1^.The conditions of ESI were as follows: 0.3 s event time, 1.5 L min^−1^ nebulizing gas (N_2_) flow rate,15 L min^−1^ drying gas (N_2_) flow rate, 350 °C interface temperature, 300 °C heat block temperature, 300 °C DL (desolvation line) temperature, 1 V DL voltage, −4.5 kV interface voltage, and 0 V Qarray DL voltage. The quantification of phenolic compounds was determined with standards curves (Table 8) of Gallic acid, Caffeic acid, Rutin, Catechin, Coumarin, Kaempferol, Apigenin, and Quercetin.

#### 3.4.3. Carotenoids Analysis

##### Total Carotenoids and Estimation of Chlorophyll

First, 200 mg of freeze-dried algae and 4 mL of crude liquid algae samples were extracted with 5 mL of acetone 80% (acetone/water: 80/20, *v*/*v*), and were sonicated for 15 min. The extraction was carried out in the dark at 4 °C. The Linchtenthaler HK (1987) Equation (2) wasused to calculate the concentration of carotenoid and chlorophyll contents [51].

Chlorophyll (a) = (12.25 A_663.2_) − (2.79 A_646.8_)
Chlorophyll (b) = (21.5 A_646.8_) − (5.10 A_663.2_)
Total Carotenoids = (1000 A_470_) − (1.82 Chlorophyll (a)) − (85.02 Chlorophyll (b)) /198 (2)

The absorbance was acquired at *λ*_470_ for carotenoids, and *λ*_663.2_ nm and *λ*_646.8_ nm for chlorophyll. The total carotenoids are expressed by milligram/gram and milligram/milliliter for freeze-dried algae and for crude liquid algae (fresh algae), respectively.

##### Carotenoid and Pigment Composition by HPLC–PDA–(ESI)–MS Analysis

The sample extracts were analyzed by using the same instrument used in Section 3.4.2. The chromatographic separation was achieved on a 250 × 4.6 mm i.d., 5.0 µm d.p.YMC30 column (YMC Europe, Schermbeck, Germany). Briefly, 20µL of the samples were injected and a binary mixture mobile phase of MeOH/acetone (60:40, *v*/*v*) (A) and ACN/water (60:40, *v*/*v*) (B) was run isocratically at 10% B for 55 min, at a flow rate of 0.8 mL/min. The LC system was coupled to an LC-qMS-2020 mass spectrometer through an APCI source (Shimadzu, Kyoto, Japan).

PDA spectra were acquired between the *λ*_220_ and *λ*_700_ nm range and chromatograms were extracted at *λ*_450_ nm and at *λ*_660_ nm for the carotenoids and the chlorophylls, respectively. MS parameters were as follows: 300–1200 *m*/*z* range, 0.2 s ion accumulation times, 2.0 L/min nebulizing gas (N_2_) flow rate, 1.05 kV detector voltage, 350 °C interface temperature, 300 °C DL temperature, 300 °C block temperature. After the identification of carotenoid compounds, the quantification was evaluated using a previously prepared β-carotene calibration curve.

#### 3.4.4. Statistical Analyses

Measures were carried out in triplicate (*n* = 3), and the results are given as mean values and standard deviations. The results were statistically analyzed using a one-way ANOVA with a statistical difference of 5% and the Tukey TSD test of the IBM SPSS software version for multiple comparisons.

## 4. Conclusions

This study evaluated the antioxidant properties of the methanolic extract of four marine microalgae, which is highly correlated with the natural antioxidant compounds present in the biomass of these microalgae. Phenolic compounds such as flavonoids, hydroxycoumarin, hydroxycinnamic acid, and hydroxybenzoic acid, and carotenoids such as ketocarotenoids, hydrocarotenoids, carotenes, and epoxycarotenoids were evaluated. A positive correlation was proved between the antioxidant activity and the polyphenol and carotenoid contents; instead, flavonoids did not show a strong correlation with antioxidant activity. In this study, *P. tricornutum*, Bacillariophyceae, isolated from the Morocco Mediterranean, produced the highest amount of carotenoids (5.14 mg/g), including all-E-fucoxanthin (35.85 μg/g dry biomass) and phenolic compounds (39.34 mg/g GAE dry biomass), represented mainly by caffeic acid (56.25 μg/g dry biomass); therefore, the best antioxidant properties (65.5%) were observed for this microalgae. *N. gaditana* showed the poorest antioxidant activity, which could be related to the lowest accumulation of carotenoids and phenolic compounds in this microalgae. Clearly, *P. tricornutum* microalgae could be suggested to be further exploited in the formulation of novel functional food or nutraceutical preparations.

## Figures and Tables

**Table 1:** Total phenolics, flavonoids, and carotenoids in the four microalgae under study.

Species	Total Phenolics (mg/g GAE)*	Total Flavonoids (mg/g QE)**	Total Carotenoids (mg/g) ^+^, (µg/mL)^++^	
***Nannochloropsis gaditana***	22.94 ^a^ ± 0.88	5.18 ^a^ ± 0.07	3.34 ^a^ ± 0.05 ^+^	1.24 ^b^ ± 0.01 ^++^
***Phaeodactylum tricornutum***	39.34 ^d^ ± 0.60	3.05 ^b^ ± 0.11	5.14 ^b^ ± 0.05 ^+^	2.09 ^d^ ± 0.01 ^++^
***Nannochloris* sp**	33.23 ^c^ ± 0.76	4.22 ^c^ ± 0.09	5.63 ^d^ ± 0.11 ^+^	1.69 ^c^ ± 0.01 ^++^
***Tetraselmis suecica***	28.03 ^b^ ± 1.17	0.61 ^d^ ± 0.08	5.62 ^c^ ± 0.12 ^+^	0.09 ^a^ ± 0.01 ^++^

Values are given as mean ± SD (*n* = 3) (absolute value). For each column, same letters indicate similar values (*p* < 0.05) * As gallic acid equivalent; ** as quercetin equivalent; ^+^ for dry biomass algae; ^++^ for crude liquid algae.

**Table 2:** Results of the antioxidant tests. DPPH test (**μ**g/mL). Reducing power (ASE/mL). Chelating activity (mg/mL).

Extract	DPPH Test IC_50_ (µg/mL)*	Reducing Power ASE/mL**	Chelating Activity IC_50_ (mg/mL)***
***Nannochloropsis gaditana***	400.00 ^d^ ± 0.01	32.71 ^d^ ± 0.02	3.52 ^b^ ± 0.18
***Phaeodactylum tricornutum***	380.00 ^b^ ± 0.01	23.98 ^a^ ± 0.11	9.69 ^c^ ± 0.32
***Nannochloris* sp**	356.00 ^a^ ± 0.01	31.48 ^c^ ± 0.05	12.82 ^d^ ± 0.04
***Tetraselmis suecica***	394.40 ^c^ ± 0.01	28.55 ^b^ ± 0.03	2.81 ^a^ ± 0.01
Standard	3.70 ± 0.17	1.443 ± 0.02	0.01 ± 3.55E − 05

Values are expressed as the mean ± SD (*n* = 3). For each column, same letters indicate similar values (*p* < 0.05). * Vit C was used as positive control, ** Ascorbic acid and BHT were used as positive control; *** EDTA was used as positive control.

**Table 3:** UV−vis, retention time, and mass spectrometry characteristics of the polyphenolic compounds tentatively identified in the four microalgae species.

Species	Peak (N°)	Rt (min)	λMax(nm)	[M − H]^−^	Fragment	Compound Identification	References
***Phaeodactylum tricornutu**m*	1	1.38	228, 260	153.1	-	Protocatechuic acid	[25]
	2	1.60	295, 324	179.2	-	Caffeic acid	[12]
	3	2.76	267, 354	683.4	341.5	Caffeic acid hexoside dimer	[26]
	4	8.85	251, 380	281.3	-	Dimethoxyflavone	[27]
	5	9.06	257	326.3	-	p-coumaroyl tyrosine	[28]
***Nannochloris* sp**	1	1.38	228, 260	153.1	-	Protocatechuic acid	[25]
	6	1.60	217, 269	277.4	-	Caffeoyl-coumaroyl-quinic acid	[29]
	7	2.53	273	289.3	-	Catechin	[30]
	4	6.92	251, 380	281.3	-	Dimethoxyflavone	[27]
	8	9.95	-	285.2	-	Kaempferol	[31]
***Tetraselmis suecica***	1	1.36	228, 260	153.1	-	Protocatechuic acid	[25]
	2	1.60	295, 324	179.2	-	Caffeic acid	[11]
	9	4.66	263, 339	341.3	-	Caffeoyl glucoside	[32]
	4	6.93	251, 380	281.3	-	Dimethoxyflavone	[27]
	5	7.28	257	326.3	-	p-coumaroyl tyrosine	[28]
	10	47.99	330	577.5	269	Apigenin-O-rutinoside	[33]
	11	49.67	249, 330, 375	611.6	594	Rhamnosyl hexosyl-methyl-quercetin	[34]
***Nannochloropsis gaditana***	1	1.39	228, 260	153.1	135	Protocatechuic acid	[25]
	2	1.60	295, 324	179.2	135	Caffeic acid	[11]
	12	3.02	247	301.2	227	Quercetin	[35,36]
	9	4.66	263, 339	341.3	323	Caffeoyl glucoside	[32]
	13	6.82	257, 360	385.4	348	Feruloylglucaric acid	[26]
	5	7.18	257	326.3	-	p-coumaroyl tyrosine	[28]
	10	47.99	330	577.5	269	Apigenin-O-rutinoside	[33]
	11	49.68	249, 330, 375	611.6	594	Rhamnosyl hexosyl-methyl-quercetin	[34]

**Table 4:** Polyphenolic components, representative of the major classes, detected in the microalgae species, along with quantitative data.

Peak	Molecules	Quantity (ppm)	Quantity (µg/g Dry Biomass)
	***Phaeodactylum tricornutum***		
1	Protocatechuic acid	6.85 ± 0.90	22.83 ± 2.99
2	Caffeic acid	16.88 ± 1.14	56.25 ± 3.81
3	Caffeicacidhexosidedimer	6.32 ± 1.13	21.07 ± 3.82
4	Dimethoxyflavone	8.51 ± 0.80	28.38 ± 2.90
5	p-coumaroyl tyrosine	4.10 ± 3.78	13.68 ± 4.58
	**Total**	42.66	113.83
	***Nannochloris* sp**		
1	Protocatechuic acid	2.26 ± 0.02	7.55 ± 0.06
6	Caffeoyl-coumaroyl-quinic acid	17.11 ± 0.52	57.04 ± 1.73
7	Catechin	10.04 ± 2.14	33.47 ± 3.14
4	Dimethoxyflavone	1.96 ± 0.16	6.53 ± 1.84
8	kaempferol	3.63 ± 0.21	12.10 ± 1.32
	**Total**	35.00	116.69
	***Tetraselmis suecica***		
1	Protocatechuic acid	12.16 ± 0.13	40.55 ± 0.44
2	Caffeic acid	17.86 ± 0.30	59.53 ± 0.98
9	Caffeoylglucoside	4.04 ± 0.35	13.46 ± 1.16
4	Dimethoxyflavone	5.7 ± 0.28	19.01 ± 1.58
5	p-coumaroyl tyrosine	5.2 ± 0.46	17.40 ± 1.55
10	Apigenin-*O*-rutinoside	10.73 ± 0.34	35.75 ± 1.13
11	Rhamnosylhexosyl-methyl-quercetin	1.38 ± 0.18	4.59 ± 1.36
	**Total**	57.07	190.29
	***Nannochloropsis gaditana***		
1	Protocatechuic acid	6.38 ± 0.96	21.26 ± 0.96
2	Caffeic acid	5.29 ± 0.21	17.64 ± 1.32
12	Quercetin	10.00 ± 0.13	33.34 ± 1.46
9	Caffeoyl glucoside	8.55 ± 0.32	28.49 ± 1.19
13	Feruloylglucaricacid	2.33 ± 0.18	7.78 ± 1.46
5	p-coumaroyl tyrosine	0.64 ± 0.07	2.12 ± 0.22
10	Apigenin-O-rutinoside	2.23 ± 0.22	7.43 ± 0.74
11	Rhamnosylhexosyl-methyl-quercetin	2.07 ± 0.16	6.89 ± 1.21
	**Total**	37.49	124.95

Mean ± standard derivation of three experiment measurements.

**Table 5:** Retention time and mass spectrometry characteristics for carotenoids tentatively identified in the four microalgae species investigated.

Species	Peaks N⁰	Rt(min)	ʎ max(nm)	*m*/*z*APCI^+^/MS	Compound Identification	Sample State
***Phaeodactylum tricornutum***	1	3.26	423, 666	545.3	Unidentified carotenoids	d.bio
	2	3.48	331, 361, 422	556.4	Unidentified carotenoids	d.bio
	3	5.77	446	659.9	All-*E*-Fucoxanthin	d.bio, c.liq
	4	6.38	333, 442	659.9	Fucoxanthin isomer	c.liq
	5	7.96	436	659.9	Fucoxanthin type	d.bio
	6	9.54	441	659.9	Fucoxanthin type	d.bio
	7	12.93	422,446,476	585.9	Diadinoxanthin	d.bio, c.liq
	8	54.61	425, 450, 478	537.9	beta-carotene	c.liq
	9	7.55	417, 442, 471	601.3	Vaucheriaxanthin	d.bio, c.liq
	10	12.01	330, 436, 463	569.9	cis-15-lutein	c.liq
	11	13.21	331,465	569.9	cis-13-lutein	c.liq
	12	14.75	442, 472	569.9	Lutein	d.bio, c.liq
	13	20.33	461	551.9	Echinone	d.bio
	8	54,59	425, 450, 478	537.9	beta-carotene	d.bio, c.liq
	14	55.38	342, 424, 446	537.9	9-cis-beta-carotene	d.bio
***Tetraselmis suecica***	15	5.76	449, 467	659.9	Fucoxanthin	d.bio, c.liq
	16	10.60	253, 345, 457, 592, 640	601.9	Violaxanthin	d.bio, c.liq
	17	11.11	345, 457, 592, 640	600.8	Cis- Prasinoxanthine	d.bio
	12	14.47	442, 472	569.9	Lutein	d.bio, c.liq
	8	54.59	425, 450, 478	537.9	beta-carotene	c.liq
***Nannochloropsis gaditana***	18	5.27	418, 438, 465	601.9	Neoxanthin	d.bio
	19	9.92	422, 444, 472	585.9	Antheraxanthin	d.bio
	20	10.75	427, 449, 477	569.9	Zeaxanthin	d.bio, c.liq
	8	54.59	425, 450, 478	537.9	beta-carotene	c.liq

d.bio: Dry biomass; c.liq: Crude liquid.

**Table 6:** Main taxonomic classification of the marine microalgae under study.

Species	Class	Phylum	Infrakingdom	Kingdom	Empire
***Phaedactylum tricornitum***	Bacillariophycea	Achrophyta	-	Chromista	Eukaryota
***Nannochloropsis gaditana***	Eustigmatophyceae	Achrophyta	-	Chromista	Eukaryota
***Nannochloris* sp**	Trebouxiophyceae	Chlorophyta	Chlorophyta	Plantae	Eukaryota
***Tetraselmis suecica***	Chlorodendrophyceae	Chlorophyta	Chlorophyta	Plantae	Eukaryota

**Table 7:** Chemical composition of modified culture medium Guillard F/2 was used as main nutrient source for marine microalgae.

Component	Molecular formula	Concentrations (mg)
Zinc sulphate	ZnSO_4_	30
Copper sulfate	CuSO_4_	25
Cobalt sulphate	CoSO_4_	30
Manganese sulphate	MnSO_4_	20
Ironchloride	FeCl_3_	50
Sodium molybdate	NaMoO_4_	25
Ethylenediaminetetraaceticacid (EDTA)	C_10_H_16_N_2_O_8_	50
Sodium nitrate	NaNO_3_	300
Sodium dihydrogen phosphate	NaH_2_PO_4_	30
Ammonium sulphate	(NH_4_)2SO_4_	20
Biotin Vit. H	C_10_H_16_N_2_O_3_S	0.1
Thiamine Vit. B1	C_12_H_17_N_4_OS	10
Cyanocobalamin Vit. B12	C_63_H_89_CoN_14_O_14_P	0.1

**Table 8:** Characteristics (UV-vis) of the phenolic standard and the corresponding LOD and LOQ values.

Compounds	UV (nm)	Regression Equation	LOQ(µg/mL)	UV LOD(µg/mL)	R^2^
Gallicacid	270	y = 3989.3x + 398.1	0.85	0.25	0.9989
Caffeicacid	321	y = 5552.1x + 4136.1	0.76	0.23	0.9983
Rutin	355	y = 1602.8x + 2741.9	2.49	0.75	0.9968
Catechin	278	y = 807.2x + 1461.2	3.25	0.97	0.9983
Coumarin	277	y = 8237.3x + 9230.6	0.67	0.20	0.9975
Kaempferol	365	y = 3481.0x + 5372.4	1.46	0.44	0.9974
Apigenin	336	y = 4915.8x − 105.3	1.16	0.35	1.0000
Quercetin	370	y = 5993.6x + 1452.1	1.29	0.39	0.9999

LOD: Limit of detection, LOQ: Limit of quantification.
